# Stem-Like Cancer Cells in a Dynamic 3D Culture System: A Model to Study Metastatic Cell Adhesion and Anti-Cancer Drugs

**DOI:** 10.3390/cells8111434

**Published:** 2019-11-13

**Authors:** Mayra Paolillo, Raffaella Colombo, Massimo Serra, Laura Belvisi, Adele Papetti, Emilio Ciusani, Sergio Comincini, Sergio Schinelli

**Affiliations:** 1 Dipartmento di Scienze del Farmaco, Università degli Studi di Pavia, 27100 Pavia, Italy; raffaella.colombo@unipv.it (R.C.); massimo.serra@unipv.it (M.S.); adele.papetti@unipv.it (A.P.); sergio.schinelli@unipv.it (S.S.); 2 Dipartimento di Chimica, Università degli Studi di Milano, 20133 Milano, Italy; laura.belvisi@unimi.it; 3 Istituto Neurologico Carlo Besta, 20133 Milano, Italy; Emilio.Ciusani@istituto-besta.it; 4 Dipartimento di Biologia e Biotecnologie ‘Lazzaro Spallanzani’, Università degli Studi di Pavia, 27100 Pavia, Italy; sergio.comincini@unipv.it

**Keywords:** metastasis, dynamic model, EMT, 3D culture, cell adhesion, integrins, SEM analysis

## Abstract

Metastatic spread is mainly sustained by cancer stem cells (CSC), a subpopulation of cancer cells that displays stemness features. CSC are thought to be derived from cancer cells that undergo epithelial to mesenchymal transition (EMT), thus acquiring resistance to anoikis and anti-cancer drugs. After detachment from the primary tumor mass, CSC reach the blood and lymphatic flow, and disseminate to the target tissue. This process is by nature dynamic and in vitro models are quite far from the in vivo situation. In this study, we have tried to reproduce the adhesion process of CSC to a target tissue by using a 3D dynamic cell culture system. We isolated two populations of 3D tumor spheroids displaying CSC-like features from breast carcinoma (MCF-7) and lung carcinoma (A549) cell lines. Human fibroblasts were layered on a polystyrene scaffold placed in a dynamically perfused millifluidic system and then the adhesion of tumor cell derived from spheroids to fibroblasts was investigated under continuous perfusion. After 24 h of perfusion, we found that spheroid cells tightly adhered to fibroblasts layered on the scaffold, as assessed by a scanning electron microscope (SEM). To further investigate mechanisms involved in spheroid cell adhesion to fibroblasts, we tested the effect of three RGD integrin antagonists with different molecular structures on cell adhesion; when injected into the circuit, only cilengitide was able to inhibit cell adhesion to fibroblasts. Although our model needs further refinements and improvements, we do believe this study could represent a promising approach in improving current models to study metastatic infiltration in vitro and a new tool to screen new potential anti-metastatic molecules.

## 1. Introduction

Over 90% of cancer-associated deaths are caused by metastatic disease, rather than primary tumors [1]. The pharmacological tools aimed at counteracting cancer growth and dissemination mainly consist of classical anti-proliferative agents like alkylating drugs, taxol derivatives, anti-hormonal therapies for hormone-dependent cancers, and, especially as a second line therapy, monoclonal antibodies. Unfortunately, these therapeutic tools often fail in the case of cancers with high metastatic tendency, also due to the occurrence of acquired resistance to chemotherapy and anti-hormonal therapy by cancer cells.

In addition, while the anti-proliferative activity of candidate drugs can be studied both in vitro and in vivo by a number of different experimental models, the metastasis formation process is more difficult to replicate in vitro. Metastatic spread processes, in fact, involve a series of sequential steps: primary tumor cells secrete extracellular vesicles (EV) that reach distant organ and tissues, are selectively taken up by non-tumor cells, and contribute to pre-metastatic niche (PMN) formation. EV can recruit different cell types to PMN and transfer oncogenic molecules between tumor cells and stromal cells in a tissue-specific way, thereby preparing the “soil” for metastasis [2,3]. A sub-population of cells originating from the primary tumor and displaying stem-like features, named cancer stem cells (CSC), acquire the ability to survive detached from extracellular matrix and from other cell contacts, and start invasion of surrounding tissue. The process continues until CSC reach blood or lymphatic vessels and intravasate [4]. Via the blood or the lymphatic stream, CSC can reach distant organs, adhere to capillary endothelial cells within the target organ, extravasate into the organ parenchyma where they proliferate, and give rise to metastatic growth.

CSC are held responsible for tumor initiation, progression and relapse, as well as metastatic disease. CSC are also thought to be responsible for the development of resistance to conventional therapies [5]. However, their origin is still debated. According to the hierarchical stem cell (SC) model of carcinogenesis, solid tumors could be generated through alterations of genetic and epigenetic factors that regulate the proliferation of SC normally present in tissues [6]. These aberrantly expressed pathways ultimately lead to the transformation of normal SC into malignant CSC. In recent years, a cellular process leading to the formation of cancer cells with stem-like features has been described, known as epithelial to mesenchymal transition (EMT). During this process, differentiated cancer cells belonging to the primary tumor, under the stimulation of a series of factors, especially TGF-β family members, undergo de-differentiation, thus acquiring stem-like features and resistance to anoikis [7].

Though the use of these cells at the moment represents the state-of-the-art of in vitro models to study metastasis-related mechanisms and anti-cancer drugs, it still poses a series of problems: the first source of CSC is the tumor specimen from which they can be isolated. Current methodologies for the isolation and study of CSC are based on the expression of surface markers specifically identified in different cancer types; CD133, in particular, has been widely utilized as a biomarker for the isolation of cells that exhibit a stem-like phenotype in a variety of cancer tissues [8]. However, CD133 is expressed by other cells present in the tumor niche and therefore, after isolation from surgical samples, a specific characterization, including stemness markers expression and in vivo tumorigenicity tests, need to be performed [9]. Due to the described technical difficulties, even though CSC clearly appear as a reliable model to study molecular mechanisms responsible for malignant cell survival and tissue invasion, the development of therapeutic strategies based on CSC-targeting has been limited. In this study, we have tried to set up an in vitro 3D dynamic model to resemble as close as possible the first steps of metastatic spread. Although this model is clearly an approximation of in vivo processes, it could be useful to investigate molecular mechanisms underlying pre-metastatic niche formation and metastatic cell adhesion. In our model, this goal could be easily achieved by cultivating classical breast and lung cell lines in specific and growth condition that select a subpopulation of breast and lung CSC without the necessity to collect CSC from tumor specimens. In addition, the demonstration that our breast and lung CSC express the majority of stemness and EMT markers, either at transcriptional or protein levels, reinforces the notion that these CSC could represent a suitable tool to investigate CSC adhesion to metastatic distant sites.

In previous studies performed in different glioblastoma models [9,10] we found that RGD integrin antagonists inhibit cancer cell adhesion, inducing anoikis in detached cells, and suggesting the potential use of these molecules in other cancer types as anti-metastatic, rather than cytotoxic agents. However, simulating at least part of the metastatic process in vitro is an unsolved issue and, eventually, in vivo models are currently used. In this study, we set up a new in vitro model to simulate the adhesion processes that cause circulating cancer cells to adhere to distant tissues, thereby initiating the infiltration process. We used millifluidic chambers connected to a medium reservoir and to a pump to simulate the lymphatic or hematic fluid flux. To resemble the 3D tissue structure of a target organ, we grew human fibroblasts on polystyrene scaffolds and maintained them in chambers for 12 days. To obtain stem-like cancer cells, we induced de-differentiation in a breast cancer (MCF-7) and a lung cancer (A549) cell line and used these cells as stem-like cancer cells.

Using this system, we tested the ability of three integrin antagonists with different chemical structures, FR-72, 1a-RGD, and cilengitide [9,11,12], to inhibit cancer cell adhesion to 3D fibroblasts, and found that only the well-known integrin antagonist cilengitide markedly inhibits CSC adhesion after 24 h. We suggest that this dynamic model could be a reliable experimental model to study metastasis-related processes, suitable for the screening of new molecules with anti-metastatic activity.

## 2. Materials and Methods

### 2.1. Compounds

1a-RGD and FR-72 were synthesized as described in previous papers [9,11]; cilengitide [12] was a gift from Prof. Laura Belvisi, Dipartimento di Chimica, Università degli Studi di Milano. These compounds, though have different chemical structures, share the common RGD (Arg-Gly-Asp) motif and are RGD-binding integrin antagonists. The compounds were solubilized in distilled water at a 2-mM concentration, and diluted in the medium to a final concentration of 20 and 50 μM for the experiments.

### 2.2. Cell Culture

#### 2.2.1. MCF-7, A549, Human Fibroblasts

MCF-7, A549, and human fibroblasts were purchased from the European Collection of Authenticated Cell Cultures. The cell lines were grown in DMEM supplemented with 10% foetal bovine serum (FBS), 2 mM L-glutamine, penicillin-streptomycin (10,000 U/mL). The cells were grown at 37 °C in a controlled atmosphere (5% CO2/95% air). Confluent cells were split (1:5–1:10 ratio, fibroblasts 1:3) by trypsinization and used at the third-fourth passage after thawing. For all the experiments, the cells were plated at a density of 10,000 cells/cm^2^. All the reagents for cell culture (DMEM, FBS, 200 mM L-Glutamine, Penicillin-Streptomycin solution (10,000 u/mL)—phosphate-buffered saline (PBS), poly-L-lysine solution, laminin, and poly-HEMA (Poly(2-hydroxyethyl methacrylate)—were purchased from Sigma-Merck (Darmstadt, Germany).

#### 2.2.2. D Fibroblast Culture

Human dermal fibroblasts were purchased from the European Collection of Authenticated Cell Cultures, ECACC (161BR, ECACC 90011810). Polystyrene scaffolds (3D Biotek Insert(Tm)-Ps Scaffold, purchased from Sigma-Merck, Darmstadt, Germany) were coated with poly-L-lysine 0.01% and laminin-1 (10 μg/mL) before the fibroblasts were plated. The fibroblasts were then grown for 10 days in millifluidic chambers, and two days before the experiments, a medium flux of 100 μL/min was activated. At the end of the experiments, the scaffolds were rinsed with PBS and placed in a 12 multi-well; cell-associated fluorescence was measured by the SynergyHT microplate reader. When SEM analysis was performed, after rinsing with PBS, the scaffolds were fixed using 3% glutaraldehyde.

#### 2.2.3. Spheroid Culture

MCF-7 and A549 spheroids were obtained by plating cells at low density (1000 cells/cm^2^) on poly-HEMA coated wells. Spheroids were growth in DMEM/Neurobasal medium (1:1) added with Glutamax (Gibco, Thermo Fisher Scientific, Waltham, MA USA), 100 μg/mL penicillin/streptomycin, 1% B-27 and N2 supplements (Gibco, Thermo Fisher Scientific, Waltham, MA USA), 10 ng/mL basic FGF and 20 ng/mL EGF (Peprotech EC, London, UK). The spheroids were maintained in culture for 21 days before the experiments and passaged at 70% confluence using Accutase dissociation reagent (Sigma-Merck, Darmstadt, Germany) and split at a 1:3 ratio. Before the experiments, the spheroids were gently dissociated, the cells were loaded by 5 μM calcein-AM (BD Italy, Milan, Italy for 30 min and then injected in the circuit.

### 2.3. Cell Viability Assays

Cell viability was assessed in millifluidic chambers using alamar blue reversible staining. The alamar blue reagent (in vitro toxicology assay kit, Rsazurin based, Tox8, purchased from Sigma-Merck, Darmstadt, Germany) is a blue, non-toxic, cell permeable, non-fluorescent compound. It is reduced by viable cells to a highly fluorescent compound that can be easily removed by a medium change. To monitor the fibroblast growth on the scaffolds, a 10× solution of the reagent was added to the culture medium and incubated for two hours at 37 °C. Fluorescence was then monitored at a wavelength of 590 nm using an excitation wavelength of 560 nm. The experiments were repeated at different times; each independent experiment was repeated three times and each medium sample was collected in quadruplicate. Results are expressed as fluorescence intensity (arbitrary unit). No variations of background values were observed during the measurements. The viability of cells injected into the circuit was checked at the end of each experiment: the cells were collected from the medium and counted; three samples for each chamber were used for the alamar blue tests.

### 2.4. Real Time qRT-PCR

To assess whether MCF-7 and A549 cells underwent EMT when grown as spheroids under our culture conditions (see above), (10) EMT markers were selected [13,14,15,16,17,18,19,20] and their mRNA expression in MCF-7, A549, and the derived spheroids was evaluated. RNA was extracted from MCF-7, A549, spheroids, and fibroblasts by Qiazol (Qiagen Italy, Milano, Italy), followed by a DNAse digestion step. RNA quality was assessed by measuring the 260/280 ratio and concentration was estimated at 260 nm. The primers were designed using the Primer3 Input software and the specificity of each primer was checked by BLAST analysis. Primers used for integrin subunits in quantitative real time RT-PCR reactions have been reported in a previous study [9] and in Table 1 the primers used to amplify EMT-related mRNAs [9] are reported in Table 2. CD44 primers were designed to recognize all the splice isoforms [19]. At the end of the reaction, a melting curve analysis was carried out to check for the presence of primer-dimers. Experiments were performed on three different cell preparations and each run was analyzed in duplicate.

Data are expressed as fold increase of each gene in spheroids compared to the relative cell line (MCF-7 and A549), using the 2^−ΔΔCt^ method [21] after normalization with the GAPDH housekeeping gene.

### 2.5. Fluorescence-Activated Cell Sorting (FACS) Analysis

The expression of αvβ3, αvβ5, α5β1, CD133, and CD44 on cell membranes was determined by FACS analysis using specific antibodies. Briefly, following gentle dissociation of the breast and lung spheres by a 1 mM EDTA/PBS solution to preserve the integrity of membrane proteins, the cells were pelleted at 800× *g* for 10 min and resuspended in 1 mL PBS to obtain a suspension of 50,000 cells/mL. Cell suspensions were then incubated with the following antibodies: Mouse monoclonal anti-integrin αvβ3 antibody (#MAB1976, Merck, Darmstadt, Germany), Alexa Fluor 633-conjugated goat anti-mouse (#A21050, Thermo Fisher Scientific), fluorescein isothiocyanate (FITC)-conjugated mouse monoclonal anti-integrin αvβ5 antibody (#MAB1961F, Merck, Darmstadt, Germany), FITC-conjugated mouse monoclonal anti-integrin α5β1 antibody (#CBL497F, Merck, Darmstadt, Germany), FITC-conjugated mouse monoclonal anti-CD44 (#NB100-63812, Novus Biologicals, Centennial, CO USA); anti-CD133 goat polyclonal antibody (#K-18, Santa Cruz Biotechnology, Dallas, TX USA) was conjugated to Atto 488 fluorescent dye using the Atto 488 Protein Labeling kit (Merck, Darmstadt, Germany) as follows: 4 μg of antibody were incubated with 2 mL of sodium bicarbonate (1 M) and with Atto 488 dye for 2 h at room temperature. Next, unbound dyes were excluded by the provided column chromatography, while the conjugated antibody was eluted in 2 mL of PBS (2 mg/mL). For FACS analysis, a 1:100 dilution of the fluorescent antibody was used.

The samples were acquired using a Beckman Coulter Navios EX flow cytometer (Brea, CA USA) with at least 10,000 events per sample and each experiment was run in triplicate. Results are reported as ratio between Mean Fluorescence Intensity (MFI) associated to bound anti-integrin or anti-CD133 and CD44 antibodies (Integrin MFI, Marker MFI) and MFI associated to isotypic controls.

### 2.6. Western Blot Analysis

The spheres were pelleted, rinsed twice in ice-cold PBS and then 200 μL cell lysis buffer (50 mM Tris/HCl, pH 7.4, 1% (*v*/*v*) NP40, 0.25% (*w*/*v*) sodium deoxycholate, 1 mM phenylmethylsulfonyl fluoride, 1 mM Na3VO4, 1 mM EDTA, 30 mM sodium pyrophosphate, 1 mM NaF, 1 mg/mL leupeptin, 1 mg/mL pepstatin A, 1 mg/mL aprotinin and 1 mg/mL microcystin) was added to the pellets. The cell suspensions were sonicated for 10 min and centrifuged at 12,000× *g* for 5 min at 4 °C. The amount of proteins in the supernatant was then determined using a Bicinchoninic Acid Protein assay kit (Pierce; Thermo Fisher Scientific, Waltham, MA, USA). For western blot analysis, 35 μg of proteins were separated by SDS-PAGE (10–12% gel) at 150 V for 2 h and blotted onto 0.22 mm nitrocellulose membranes at 50 mA for 16 h at 4 °C. The membranes were first blocked for 2 h in Tris-buffered saline containing Tween-20 (TBST; 10 mM Tris/HCl, 150 mM NaCl and 0.1% Tween-20) containing 4% non-fat dry milk powder (TBSTM) and then incubated with the appropriate antibody (SNAI1: #3879S, Cell Signaling, Danvers, MA; SNAI2, #9585S, Cell Signaling; SOX2, #ab92494, Abcam, Cambridge, UK; N-Cadherin, #610920, BD Transduction Lab, Italy, Milan, Italy; E-Cadherin, #610182, BD Transduction Lab Italy, Milan, Italy; Vimentin, #NCL-VIM clone VIM 3B4, Leica Biosystem, Wetzlar, Germany; Tubulin, #2144S, Cell Signaling, Danvers, MA) diluted 1:1000 in TBST-4%BSA at 4 °C for 16 h with gentle agitation. The membranes were rinsed three times in TBST and then incubated at 21 °C for 2 h with horseradish peroxidase-conjugated secondary antibodies (anti-rabbit IgG, HRP-linked antibody #7074, Cell Signalling, Danvers, MA; anti-mouse IgG, HRP-linked antibody #7076, Cell Signalling, Danvers, MA) diluted 1:10,000 in TBST-BSA. The membranes were rinsed three times in TBST and the luminescence signal was captured using an ImageQuant LAS 4000, GE Healthcare. Each experiment was performed in triplicate.

### 2.7. Dynamic Bioreactor Set-Up and Conditions

**Figure 1:**
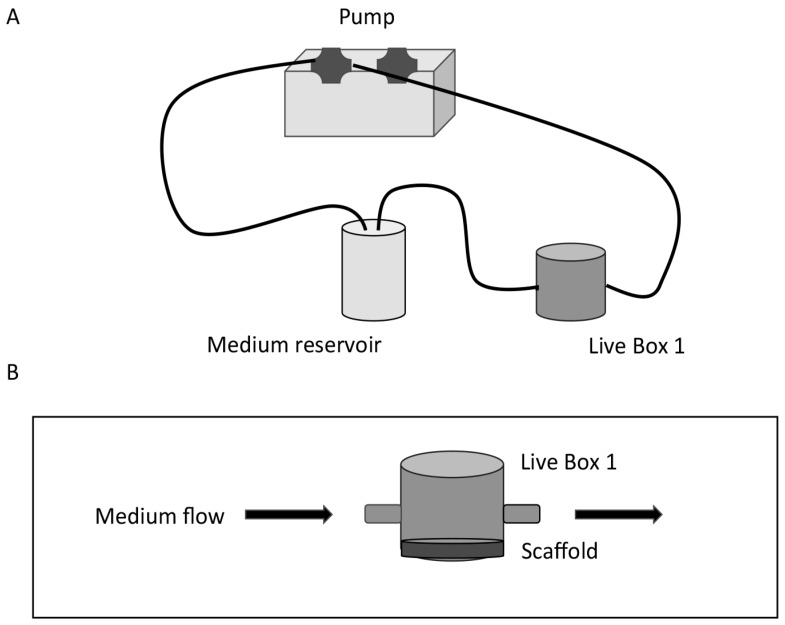
Schematic diagram of the set-up of a dynamic bioreactor. (**A**) The Live Box 1 (LB1) millifluidic chamber is connected to a medium reservoir and the medium flow (100 μL/min) is regulated by a pump. (**B**) Details of LB1 with the scaffold placed on the bottom of the chamber and tangential medium flow.

The multi-compartmental modular bioreactors, LB1 and LB2 (LiveFlow^®^ system), were purchased from IVTech (IVTech, LU, Italy); LB1 is a 24-well sized transparent milli-scaled chamber for fluidic culture of scaffolds and membranes under low shear stress, while LB2 is designed to allow two combinations of flow inputs and outputs. This system was previously used to mimic different tissues and organ compartments [22]. The LB2 chamber was used in preliminary experiments; LB2 is equipped with two flow inlets and outlets and a holder to house a porous membrane where scaffolds can be placed with a cross-flow top-down configuration, according to the manufacturer’s instructions. Afterword, since fibroblasts did not appear to properly adhere to the scaffolds, the LB1 chamber was chosen for our experiments. The scaffolds were placed on the bottom of the chamber and fibroblasts were maintained in culture for 12 days before starting the experiment. The medium flow, with a tangential configuration (Figure 1), started 48 h before the experiments. Twenty-four hours before stem-like cancer cells derived from dissociated spheroids were added, the fibroblasts’ growth medium was replaced by the serum-free spheroids medium (see cell culture paragraph) and eventually, the dissociated cancer cells from the spheroids were added to the circuit. The total circuit volume was 12 mL, the flow rate was set at 100 μL/min since, in previous experiments, we observed that higher flow rates did not fully allow cell adhesion on our scaffolds.

Finally, the LiveFlow^®^ system was placed in the cell culture incubator under standard cell culture conditions for 24 h.

### 2.8. SEM Analysis

Microstructural characterization was performed with a high-resolution scanning electron microscope (TESCAN, Mira 3 XMU) equipped with an In-Beam SE detector and operated at 8 kV. Samples were fixed by a 2.5% glutaraldehyde solution for 3 h at 4 °C and then coated with platinum using a Cressington 208HR (60 s deposition, 20 mA).

### 2.9. Fluorescence Analysis

To quantify stem-like cancer cells attached to the fibroblast coated scaffolds under dynamic flow conditions, in the presence or in the absence of integrin antagonists, the stem-like cancer cells were loaded with 5 μM calcein-AM (BD Biosciences) for 30 min before being injected into the circuit. At the end of the experiments, the cells remaining in the medium were subjected to cell viability tests by the alamar blue kit (see cell viability paragraph), while the fluorescence associated to the cells on the scaffolds was measured by a Synergy HT microplate reader (Biotek, Winooski, VT USA) at 590 nm with an excitation wavelength of 560 nm. As an additional control, in some experiments, the scaffolds after fixing by glutaraldehyde were observed by a fluorescence microscope.

### 2.10. Statistical Analysis

Data are expressed as mean ± standard deviation (SD) and analyzed using Student’s *t* test when comparing the two groups. To compare more than two groups, we used one-way ANOVA and post hoc Tukey tests. P values were considered significant when <0.05 and denoted as follows: * *p* < 0.05. Statistical analyses were performed using the GraphPad Prism software (version 5.0).

## 3. Results

### 3.1. Set Up of the Dynamic In Vitro 3D Culture System

**Figure 2:**
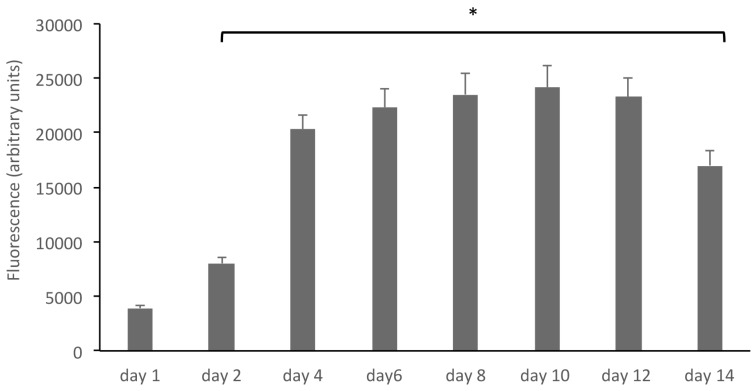
Human fibroblasts grown on polystyrene scaffolds grow and proliferate. The growth of human fibroblasts in 3D was assessed by the alamar blue reagent (Tox8, resazurin), added to the culture media at increasing times. Results, expressed as fluorescence intensity (arbitrary unit), show an increase of cell viability up to 12 days. * *p* < 0.05 compared to controls (day 1).

Fibroblasts were grown on polystyrene scaffolds in LB1 chambers and at day 10 before the experiments were shifted to dynamic culture conditions with a medium flux of 100 μL/min for other 48 h. Under these conditions, cell viability experiments were performed by the alamar blue test (see methods); briefly, the alamar blue reagent (Tox8, resazurin) was added to the culture media at increasing times and fluorescence was read at an excitation wavelength of 590 nm. Results, expressed as fluorescence intensity (arbitrary unit), are summarized in Figure 2. An increase of cell viability is observed up to 12 days, while on day 14, a reduction is scored.

Fibroblasts in culture are known to synthesize and release fibronectin (FN), especially when subjected to peculiar growth conditions [23]; for this reason, as a functional correlate, the FN1 mRNA content was measured on day 6 and day 12 in fibroblasts grown in 2D and 3D, by quantitative real-time RT-PCR: on day 6, a 1.23 ± 0.2 fold increase in 2D and 2.52 ± 0.4 fold increase in 3D was found; on day 12, a 9.99 ± 1.6 fold increase in 2D and a 19.2 ± 2.1 fold increase in 3D, compared to fibroblasts in culture after 24 h, was observed (Table 3).

**Figure 3:**
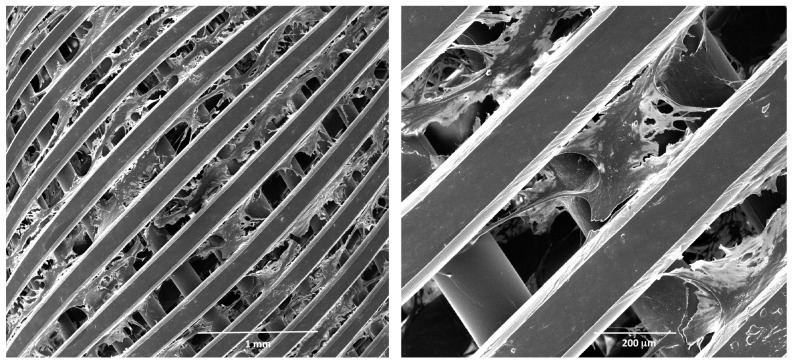
Scanning electron microscopy (SEM) analysis of human fibroblasts grown in 3D. Micrographs at different magnifications of fibroblasts grown on a polystyrene scaffold on day 12 in culture. Before SEM analysis, the scaffolds were fixed in glutaraldehyde and coated by platinum.

Finally, the fibroblast grown in 3D was also assessed by scanning electron microscopy, as described in the Methods section. In Figure 3, micrographs at different magnifications of a scaffold coated by fibroblasts on day 12 in culture are reported. These data, overall, show that fibroblasts grown in 3D under dynamic conditions proliferate, produce fibronectin, and form a healthy 3D architecture, suitable to mimic target tissue structure in vitro up to 12 days.

### 3.2. MCF-7 and A549 Cancer Cells Acquire a Stem-Like Phenotype

**Figure 4:**
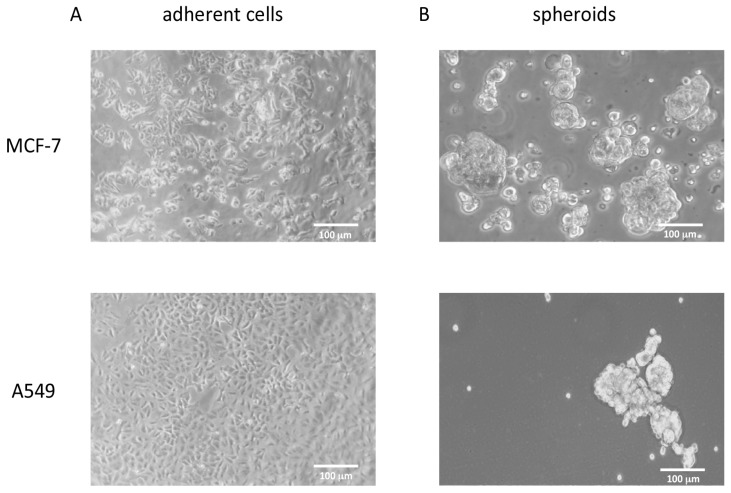
Phase contrast pictures of MCF-7 and A549 cell lines grown under different conditions. (**A**) MCF-7 and A549 cell lines grown as monolayer in the presence of 10% FBS. (**B**) The same cells grown in suspension as spheroids under de-differentiating conditions.

MCF-7 and A549 cells were grown in poly-HEMA coated wells in DMEM/neurobasal medium (1:1, see Methods) for 21 days. Under these growth conditions, the cells acquired the ability to grow in suspension, duplicate, and form spheroids of non-adherent cells that can be propagated, suggesting that a de-differentiation process underlies these morphological changes (Figure 4).

We evaluated TGF-β mRNA in MCF-7, A549, and in the relative spheroids to assess whether in these cells, grown under the described conditions, TGF-β could promote a de-differentiation process by an autocrine mechanism. We found a significant increase of TGF-β mRNA in spheroids compared to the differentiated cells, confirming our hypothesis, and suggesting that spheroids, by synthesizing and then releasing TGF-β could prime the EMT process (Table 4).

To assess whether the MCF7 and A549-derived spheroids displayed differences in stem marker expression compared to MCF7 and A549 cells grown under classical differentiating conditions, i.e., in the presence of serum as adherent cells, the transcriptomic analysis of 10 stemness markers related to EMT (CD133, CD44, ZEB-1, SOX-2, E-Cadherin, N-cadherin, Vimentin, Fibronectin-1, SNAI-1, SNAI-2) [13,14,15,16,17,18,19,20,24] was performed by quantitative real-time RT-PCR.

**Figure 5:**
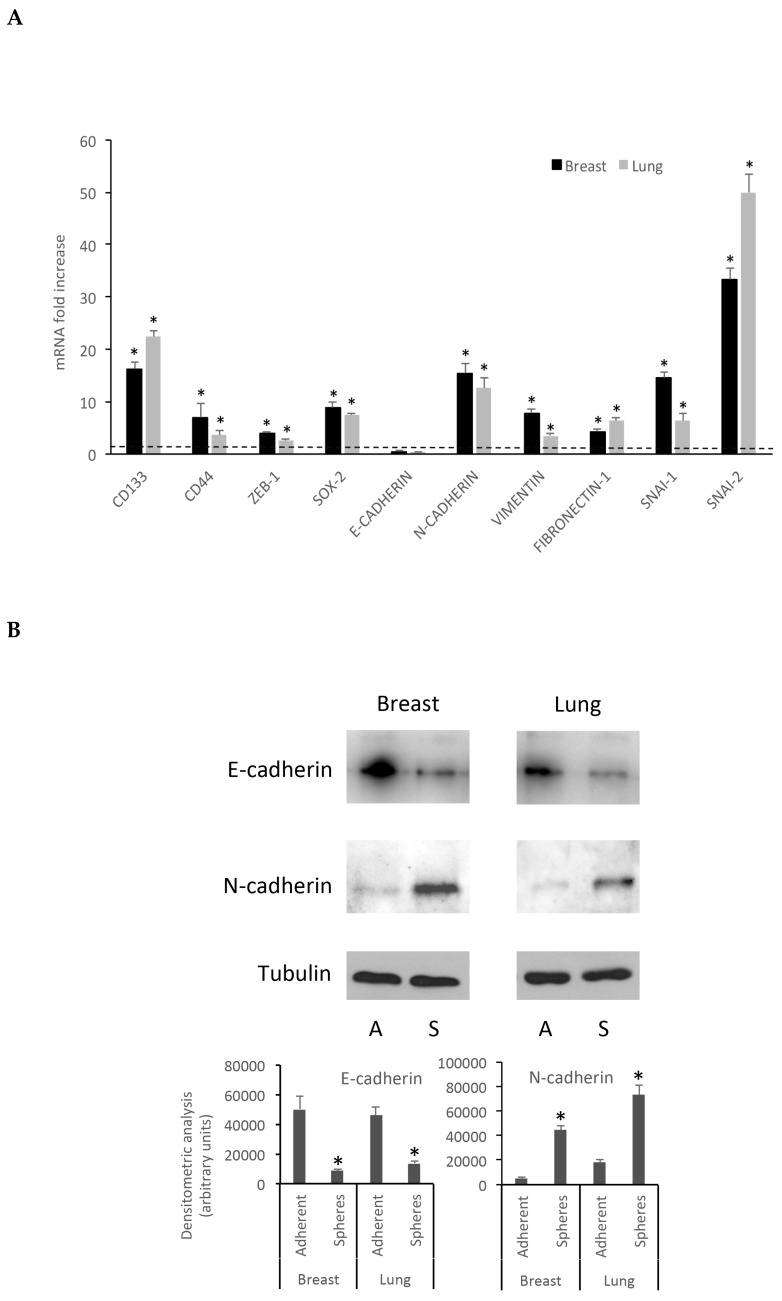
Breast and lung spheroids express EMT-related genes mRNA. (**A**) The mRNA expression of 10 EMT-related genes (CD133, CD44, ZEB-1, SOX-2, E-Cadherin, N-cadherin, Vimentin, Fibronectin-1, SNAI-1, SNAI-2) was observed in breast and lung spheroids compared to the corresponding MCF-7 and A549 cell lines grown as monolayer, by quantitative real-time RT-PCR. Data are expressed as fold change, calculated by the ΔΔ-Ct method. Dotted line: fold increase = 1. * *p* < 0.05 compared to controls (MCF-7 and A549 grown as monolayers, under differentiating conditions). (**B**) Spheroid cell protein extracts were subjected to electrophoresis and western blot: 35 μg of proteins were loaded per lane and then processed for western blot as reported in the Methods section; A: adherent cells; S: spheres.

qRT-PCR data, reported in Figure 5A, are expressed as fold increase, calculated by the ΔΔ-Ct method [21], using *GAPDH* as a reference housekeeping gene. In qRT-PCR experiments, the EMT marker transcripts displayed a significantly higher expression in spheroids rather than in the corresponding differentiated cells, with the exception of E-cadherin, whose mRNA expression does not significantly differ between MCF7 and spheroids. Interestingly, a decrease of E-cadherin and a marked increase of N-cadherin mRNA expression was observed in both spheroid cell populations: this striking feature suggests that, under the described culture conditions, the cells undergo the cadherin switch, observed during EMT transition [25,26], thereby acquiring a stem-like phenotype. These data were further confirmed by western blot and FACS analysis of the relative proteins showing the “cadherin switch” in both breast and lung spheroids (Figure 5B) together with changes in the expression of other EMT and stemness markers (Figure 5B and Supplementary Material), in good agreement with qRT-PCR data. CD133 and CD44 expression on the cell surface, evaluated by FACS analysis, was in good agreement with qPCR data (Supplementary Material).

We named these spheroids breast and lung spheres, and cells obtained from the spheroids by dissociation were named CSC-like cells; they will be referred to in this way in the text.

### 3.3. Breast and Lung Spheroids Express αv, α5, β1, β3, β5 Integrin Subunits

**Figure 6:**
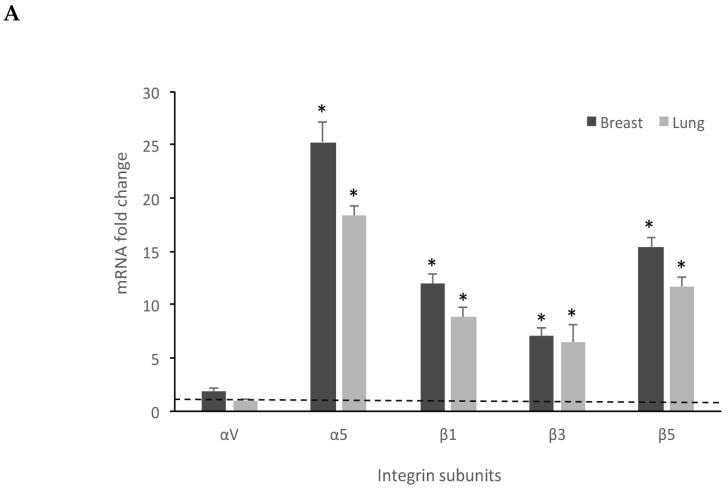

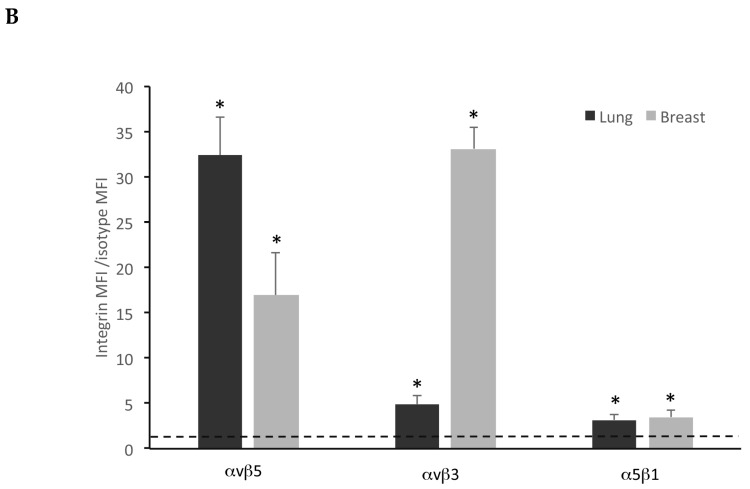
Breast and lung spheroids express integrin subunits. (**A**) Breast and lung spheroids, compared to the corresponding MCF-7 and A549 cell lines grown as monolayer, display an increased mRNA expression of α5, β1, β3, β5 integrin subunits, measured by quantitative real-time RT-PCR, while αv mRNA appears not to significantly vary in both breast and lung spheroids. Data are expressed as fold change, calculated by the ΔΔ-Ct method. Dotted line: fold increase = 1. * p < 0.05 compared to the controls (MCF-7 and A549 grown as monolayers, under differentiating conditions). (**B**) The expression of integrin receptors on the cell surface was assessed by FACS analysis: both lung and breast spheroid-derived cells express αvβ5, αvβ3, α5β1 integrin receptors. Data are expressed as ratio between mean fluorescence intensity associated to bound anti-integrin antibodies (Integrin MFI) and mean fluorescence intensity associated to isotypic controls. Dotted line: expression threshold = 1. * p < 0.05 compared to isotypic controls.

Previous reports have demonstrated that some RGD-binding integrins are overexpressed in solid tumors [27] and integrin expression in stem or stem-like cancer cells plays complex roles in cancer progression [28]. Therefore, we sought to evaluate the αvβ3, αvβ5, and α5β1 integrin expression pattern in breast and lung spheres by measuring the mRNA amounts of αv, α5, β1, β3, and β5 subunits, with the aim of investigating the adhesion of breast and lung spheres to a substrate. Our qRT-PCR experiments clearly showed that all the subunits are over-expressed in the spheres compared to the differentiated cancer cells (Figure 6), with a marked increase of α5 (25.28 ± 1.9 fold increase in breast spheres and 18.39 ± 0.9 in lung spheres), β1 (12.01 ± 0.9 fold increase in breast spheroids and 8.86 ± 0.87 in A549 spheroids), β3 (7.06 ± 0.8 fold increase in breast spheroids and 6.42 ± 1.7 in A549 spheroids), and β5 (15.35 ± 1.01 fold increase in breast spheres and 11.72 ± 0.85 in lung spheroids), thus identifying these cells as a suitable model to study integrin-mediated adhesion processes.

FACS experiments, using fluorescent-conjugated antibodies recognizing αvβ3, αvβ5, and α5β1 integrins, confirmed the expression of integrins on spheroid-derived cell membrane (Figure 6B and Figure S1 Supplementary Materials). Results are reported as ratio between mean fluorescence intensity associated to anti-integrin antibodies (Integrin MFI) and mean fluorescence intensity associated to isotypic controls.

### 3.4. Integrin Antagonists Inhibit Adhesion of Stem-Like Cells

Once the system was settled, the ability of integrin antagonists to inhibit cell adhesion under dynamic conditions was tested. Three RGD-containing molecules were tested, the first and well-known cyclic pentapeptide cilengitide [12] and two peptido-mimetics, FR72 and 1a-RGD [10,11]. These compounds were tested on both breast and lung spheroids at 20 and 50 μM concentrations under the experimental conditions described above. The concentrations were comparable with those used in previous studies [10,11]. Briefly, 24 h before the experiments, the fibroblast medium was substituted by the DMEM/Neurobasal medium used to grow spheroids. After that, breast and lung spheres were gently, mechanically dissociated, counted, and incubated with calcein-AM 5 μM for 30 min; at the end of this incubation, the cells were injected into the circuit, where the medium was circulated at 100 μL/min and the circuit was placed into the cell incubator for 24 h. At the end of the experiments, still floating cells were recovered, counted, and cell viability was tested by alamar blue. The scaffolds were rinsed by PBS and the fluorescence associated to calcein-loaded cells adherent to the scaffolds was measured by a microplate reader.

**Figure 7:**
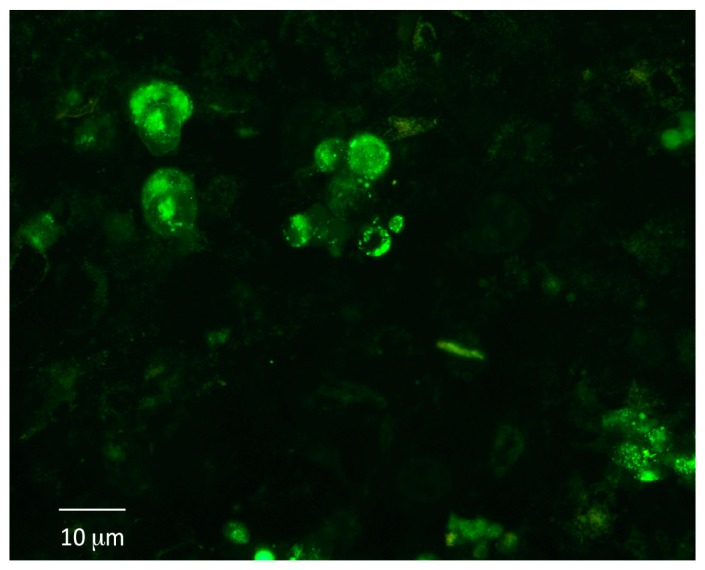
Adhesion under dynamic conditions of breast stem-like cancer cells to fibroblasts grown in 3D. Stem-like cancer cells, obtained by gentle mechanic dissociation from breast and lung spheroids, were loaded with calcein-AM and injected into the circuit. After 24 h, calcein-loaded cells were detected on fibroblast-coated scaffolds.

For fluorescence microscope or SEM analysis, after rinsing, the scaffolds were fixed by glutaraldehyde and eventually coated by platinum (see Methods) (Figure 7). When integrin antagonists were used, the compounds were injected into the circuit at proper concentrations for 1 h before injecting the cells, except for controls, and then the experiment was carried out as described above.

**Figure 8:**
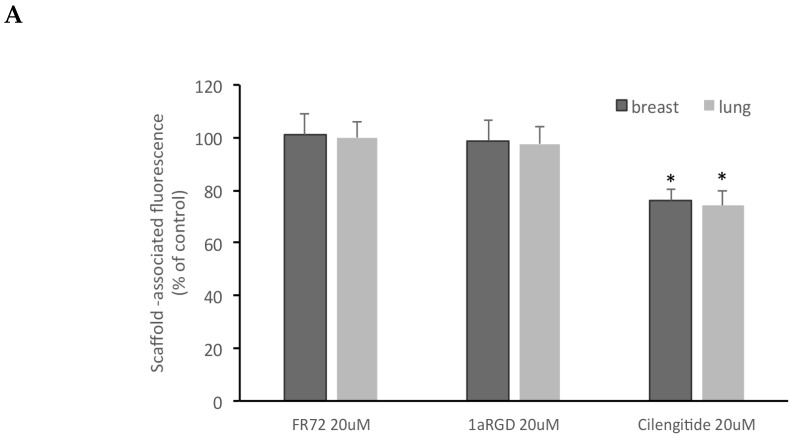

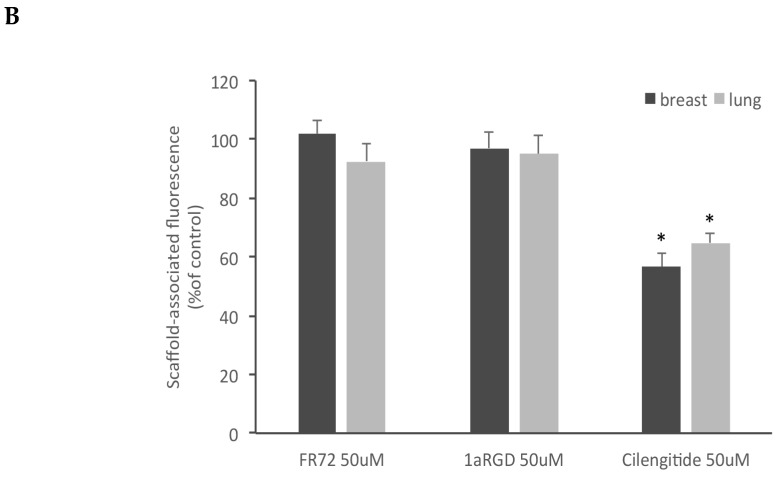
Cilengitide inhibits stem-like cancer cells adhesion to fibroblasts grown in 3D. Three RGD-containing integrin antagonists, FR72, 1a-RGD, or cilengitide, were injected in the circuit at 20 (**A**) or 50 μM (**B**) concentrations and stem-like cancer cells’ adhesion to fibroblast-coated scaffolds was evaluated by measuring scaffold associated fluorescence. Only cilengitide inhibited cell adhesion at both concentrations. * p < 0.05 compared to the controls.

Of the three compounds tested, after 24 h contact, cilengitide was able to inhibit adhesion of breast and lung stem-like cells to scaffolds at 20 and 50 μM (Figure 8), while FR72 and 1aRGD were apparently ineffective in preventing cell adhesion at both concentrations.

**Figure 9:**
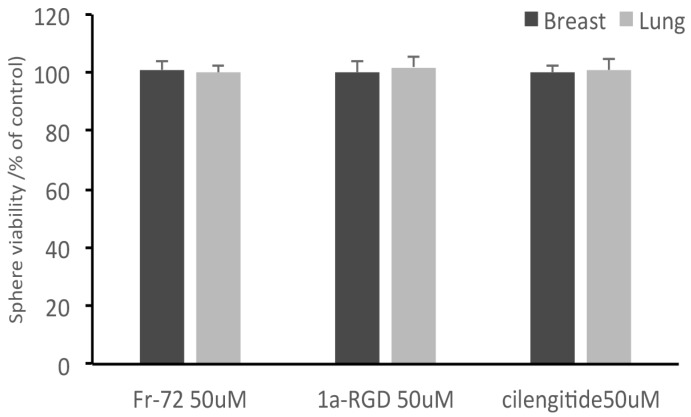
Integrin antagonists do not affect stem-like cancer cell viability during the treatments. At the end of the treatments, stem-like cancer cells were recovered from the circuit and subjected to alamar blue cell viability tests. None of the three compounds was found to affect cell viability after 24 h contact.

Concurrently, no decrease of cell viability was observed in the cells recovered from the circuit, even in the presence of FR72, 1aRGD, and cilengitide (Figure 9); these data showed that no toxic effect was exerted by the compounds and indicated that the decreased fluorescence signal measured in the scaffolds was due to the anti-adhesive properties of cilengitide rather than to an aspecific cytotoxic effect.

## 4. Discussion

The metastatic process is a complex and dynamic multi-step mechanism involving several different cell types. This scenario makes the study of metastasis a daunting task; indeed, in the past, several drug candidates failed in translation from in vitro experimental models to in vivo tests. Therefore, in this field, there is an urgent need to implement reliable in vitro models that could mimic as close as possible the intrinsic complexity of the in vivo metastatic conditions. With the aim to improve the experimental systems in this field, in this work, we have set up a reliable and versatile model to investigate the interplay among the signaling pathway implicated in the attachment of circulating tumor cell to a solid scaffold previously coated with non-tumor “target cells”.

The original goal of our study dealt with the idea to implement a complex in vitro 3D model based on the co-culture of endothelial cells with fibroblasts to mimic the vascular structure. Indeed, in the intravasation process, circulating tumor cells (CTCs) must initially attach to endothelial cells and engage a direct and tight interaction with cells associated to the basement membrane, particularly fibroblasts, to reach the underlying stroma and colonize distant organs [29].

However, our preliminary attempts aimed at layering and growing endothelial cells on our scaffold, alone or in combination with fibroblasts, were unsuccessful. In addition, alternative approaches to solve this issue by pretreating our scaffold with matrigel or ECM-like mixture did not lead to any significant improvements.

Although we are aware that an in vitro 3D scaffolded model based only on a single cell population is a very simplified representation of any actual in vivo scenario of target tissue, fibroblasts play key roles in shaping the microenvironment that contributes to pre-metastatic niche formation.

In epithelial cancers, stromal fibroblasts (together with other stromal cells such as endothelial cells and adipocytes, etc.) promote EMT, tumor initiation and progression, and regulate deposition and remodeling of ECM proteins in tumor niche and tumor-surrounding environments [30].

Notably, growth factors and chemokines, produced by cancer and endothelial cells, promote stromal fibroblast activation, and differentiation into myofibroblasts and cancer-associated fibroblasts (CAFs) [31], which, in turn, synthesize and release significant amounts of ECM proteins, thus modulating CTCs/niche interactions.

Lastly, fibroblasts can be converted in osteoblasts by ALK5 inhibitors and other treatments [10], highlighting the exciting possibility to further improve this approach aimed at obtaining a reliable bone metastasis model.

For all these reasons, we believe that the experimental model described in this work, with future improvement and additional refinement, may represent a versatile starting point to implement an in vitro model potentially useful for drug screening and to gain further insights into the molecular mechanisms involved in cell-cell interactions.

Although cell lines grown in the monolayer provide a useful and homogenous model, several studies have clearly demonstrated that cells cultured in 3D display features that better mimic in vivo conditions [32,33], thus partially filling the gap between in vitro studies and in vivo applications. More than 380 cell lines have been studied using 3D culture methods in different fields such as cancer research, cell growth, apoptosis, survival, cell differentiation, gene, protein expressions, drug discovery, and cytotoxicity [32].

Another important mechanism likely involved in the adhesion of CSC to fibroblast could require the involvement of extracellular vesicles (EV). EV are known to play an important role in priming target tissue cells to initiate the pre-metastatic niche formation process via the transfer of their cargo molecules, such as oncoproteins, oncopeptides, receptors, adhesion molecules, microRNAs, mRNAs, lipids, and DNA fragments to recipient target cells, thereby promoting dramatic phenotypic changes [34]. Although in our study we have not investigated the putative role of EV in adhesion of CSC to fibroblast, we believe that a dynamic flow model could be particularly suitable to study the effect of EV in educating recipient cells and thus preparing suitable soil for the CSC adhesion. Due to the relevance of this mechanism, we plan to investigate the role of CSC-derived EV in future experiments.

The use of rigid polymer scaffolds to support the structure of 3D tissue models has been described for a variety of applications and tissue types [35,36]; these scaffolds promote cell adhesion and their pores allow the migration of seeded cells under perfusion by a dynamic flow-through system.

Fibroblasts physiologically tend to fill spaces within tissues and form ECM and, as shown by SEM micrographs, form a 3D network that could be particularly suitable to study ECM-CTC interactions. Indeed, CTCs’ attachment to seeded cells is mediated by several mechanisms such as podosomes, point contacts, and focal contacts that require integrin activation [37]. The role of integrins is especially critical because they link the external ECM to the cell intracellular actin cytoskeleton; therefore, interfering with these attachment mechanisms could be a useful tool to inhibit CTCs’ adhesion to target tissues. A dynamic medium flow-through system, providing a constant oxygen supply and stable gradients of medium components, allows better growth and survival conditions in comparison to a static model. The most critical parameter to be carefully evaluated in this models is flow rate; the choice of the most appropriate value for this parameter is further complicated by the relative paucity of published studies, performed in similar or analogous models dealing with this issue. The features of dynamic flow systems have been particularly studied in 3D microfluidic models in which a positive relationship between flux rate and tumor dimensions was reported [38]. On the basis of flow rate (50 μL/min) used in this study, we assumed that in our experimental model, taking into account the structural differences, a 100 μL/min could be reasonably adopted. The ideal flow rate is a subtle compromise between opposite flow-rate-dependent effects; high flow rates decrease the time duration of the interaction between CSCs and cells deposited on the scaffold, thus lowering the amount of CSCs attached to fibroblasts. Conversely, low flow rates increase the risk of aspecific binding of CSCs to system components and raise the chance of forming microaggregates, thus undermining the advantage of a dynamic system over classical 2D models. However, we are very well aware that our experimental conditions are based on empiric assumptions to be further tested and validate by future studies.

Our data show that fibroblasts grown in 3D on polystyrene scaffolds proliferate and synthesize fibronectin, creating a 3D structure that could resemble a tissue structure. In future experiments, human osteoblasts will be used to simulate a bone structure to specifically study bone metastasis mechanisms.

However, our experimental model suffers from several drawbacks that need to be carefully addressed. Beyond the already discussed issue of evaluating the effects of EV in our model, other limitations are represented by the contact time of breast and lung CSC to scaffolded fibroblasts and by the consideration that different cell types beyond fibroblasts belonging to the tumoral niche, such as endothelial cells and macrophages, contribute to modulate the adhesion of putative metastatic CSC to distant sites.

One of the key steps in metastatic cascade is the migration of tumor cells from the primary tumor to other distant sites. The ability of cells to migrate from one location to another is an important feature in several normal processes, like development and tissue regeneration, and this migratory capacity is largely due to an EMT process [39]. It has been hypothesized that CSC, generally held responsible for metastatic invasion, may also activate their migration through EMT [40]. Isolating CSC from human tumor samples is a complex practice that, in addition, requires CSC characterization in vitro and in vivo [9]; a valuable alternative is represented by CSC-like cells derived from differentiated cancer cells [41]. In this study, we obtained CSC-like cells from differentiated breast and lung cancer cells by plating them at low density and growing them under the same conditions used for stem cells (see Methods). They acquired the ability of growing in suspension, forming spheres that could be dissociated and propagated. To test whether these cells acquired stem-like features, we selected a set of EMT markers on the basis of previous studies reported in the literature [13,14,15,16,17,18,19,20]. After three weeks in culture, we found an increase of these EMT markers mRNA and a marked increase of N-cadherin in both breast and lung spheres, compared to the differentiated counterparts; these data, confirmed by our western blot and FACS experiments, strongly indicate that in our experimental conditions, the cell underwent EMT. In addition, to further support this hypothesis, in parallel experiments (not shown) we found that our CSC-like cells expressed TGF-β mRNA, suggesting that EMT could be induced by TGF-β synthesized by the same cells.

Tumor progression, invasion, and eventual metastasis require the activity of a variety of adhesion proteins, including the integrin superfamily. At each stage of cancer progression, subsets of integrin heterodimers are activated, providing the necessary signaling pathways for adhesion, migration, and cell survival. Metastatic tumor cells show differential integrin heterodimerization and activation compared to non-metastatic tumor cells that enable the cell to home and colonize in a metastatic site [42]. Many integrins have been implicated in tumor cell-host tissue interactions, including the β1, β3, and β5 integrin family members [42].

We found an increased mRNA expression for β1, β3, β5, and α5 integrin subunits in breast and lung stem-like cells, compared to the relative differentiated cell line. The concomitant surface expression of these integrins by FACS analysis showed an apparent discrepancy in αvβ5 expression, as measured by FACS, compared to qPCR data; the experiment was repeated using different cell preparations, but the results did not significantly change. This apparent discrepancy could be due to the antibody binding affinity for the integrin dimer and, in addition, it should be considered that qPCR data are expressed as fold increase in comparison to differentiated MCF-7 and A549 cell and cannot be considered an absolute mRNA quantification. However, the integrin receptor expression was confirmed by FACS analysis, supporting the notion that our approach could represent a novel and interesting model to study anti-adhesion molecules like RGD-based integrin antagonists. In previous studies, we found that 1a-RGD and FR-72, two RGD integrin antagonists, inhibited cell migration and focal adhesion kinase (FAK) phosphorylation [9,11]. FAK is a serine-threonine kinase downstream αvβ3, αvβ5, and α5β1 integrin receptor that is phosphorylated upon binding to fibronectin, vitronectin, and other ECM components containing the RGD motif. Cilengitide is the first RGD integrin antagonist synthesized by Kessler and his research group [12]. Cilengitide reached phase III in clinical trials and aroused many hopes, especially for high-grade cancers, but no significant advances were found at the end of these studies [43,44]. In this work, we have tested the anti-adhesive properties of these molecules by injecting them into the circuit to inhibit CSC-like cell adhesion to fibroblasts-coated scaffolds at 20 and 50 μM concentrations. We found in both cases that only cilengitide was able to significantly inhibit cell adhesion. These results may be explained considering that in our previous studies 1a-RGD and FR-72 were kept in contact with cancer cells, under classical static in vitro conditions, for 48 h and only after this time period was cell detachment observed. Cilengitide, instead, induced cell detachment after 24 h (unpublished observations). Unfortunately, under our conditions, we could not extend our exposure time because after 24 h calcein content in CSC-like cells was drastically reduced with fluorescence signals no longer detectable.

In previous works, RGD antagonists were used at 20–25 μM concentrations under static conditions in 2D cultures and the molecules were added daily up to 72 h [9,10,11]; under these experimental conditions, cell detachment induced anoikis without any sign of direct cell toxicity. The lack of cell toxicity found in several in vitro models is in excellent agreement with clinical studies that have tested the effect of cilengitide. Indeed, in clinical trials, cilengitide administered at considerably high doses (2000 mg intravenously twice weekly over 1 h infusion) and for long time periods (up to 77 weeks) [CENTRIC trial, ClinicalTrials.gov Identifier: NCT00689221; ClinicalTrials.gov Identifier: NCT01118676], displayed no serious toxic events. For these reasons, although we cannot exclude possible toxic effects induced by these integrin antagonists on other cell types in vivo, we were unable to find any toxic effects under our experimental conditions in CTCs. In addition, both 1a-RGD and FR-72 display in vitro high affinity for αvβ3 (1a-RGD IC_50_: 6.4 ± 0.1 nM, against echistatin; FR-72 IC_50_: 4.5 ± 1.1 against vitronectin) and lower affinity for α5β1 (1a-RGD IC_50_: > 500 nM, against echistatin; FR-72 IC_50_: > 500 nM, against vitronectin) [45,46]. Cilengitide, on the other hand, displays high affinity for both αvβ3 and α5β1 (αvβ3 IC_50_: 0.61 ± 0.06 nM, against vitronectin; α5β1 IC_50_: 14.9 ± 3.1 against fibronectin) [47], and we suggest that integrin expression pattern in different cell types could also account for the results obtained with these molecules. These results suggest that cilengitide and other RGD integrin antagonists could have proper therapeutic applications as anti-metastatic drugs, rather than cytotoxic drugs, to prevent metastatic cell colonization: the fact that cilengitide failed in clinical trials when given to high grade cancer patients, in association with cytotoxic drugs, was not unexpected, considering that the most striking feature of this molecule is the ability to inhibit cell adhesion, even under dynamic conditions.

In conclusion, in this work, we propose a 3D dynamic model to study metastasis processes, such as the formation of premetastatic niche and attachment of CTCs. Although we are very well aware of the intrinsic limitations and challenges associated with our model, we believe it could help scientists in the field to improve existing in vitro models and to implement future dynamic in vitro systems devoted to investigating metastatic processes. In addition, by adding directly into the circuit-purified EV, our model could exploit the role of EV released by primary tumors cells and/or by CTCs in priming several types of target non-tumor cells grown on our 3D scaffold.

## Figures and Tables

**Table 1:** Primers used to amplify RGD-binding integrin mRNA in qRT-real-time PCR experiments.

GENE	ACCESSION NUMBER	PRIMER SEQUENCE
αv	NM_002210	F: actggcttaagagagggctgtg R: tgccttacaaaaatcgctga
β3	NM_000212	F: tcctcaggaaaggtccaatg R: tcctcaggaaaggtccaatg
β5	NM_002213	F: agcctatctccacgcacact R: cctcggagaaggaaacatca
α5	NM_002205	F: cctgctgtccaccatgtcta R: ttaatggggtgattggtggt
β1	NM_133376	F: tccaatggcttaatttgtgg R: cgttgctggcttcacaagta
GAPDH	NM_002046.5	F: cagcaagagcacaagaggaag R: caactgtgaggaggggagatt

**Table 2:** Primers used to amplify EMT*- and stemness^-related genes in qRT-real-time PCR experiments.

	ACCESSION NUMBER	PRIMERS SEQUENCE
CD133^	NM_006017.2	F: ccaccgctctagatactgctg R: cctatgccaaaccaaaacaaa
ZEB-1^	NM_030751.5	F:tgcctttctgactctcagctc R:cctgttaggcagtgaggaatg
SOX-2^	NM_003106	F: ggacttctttttgggggacta R: gcaaacttcctgcaaagctc
E-CADHERIN*	NM_004360.3	F: gcaggagagcttgtcattgac R: agactcctccattccttccag
N-CADHERIN*	NM_001792.4	F: acagctccaccatatgactcc R: tcctgctcaccaccactactt
VIMENTIN*	NM_003380.3	F: ccaagtttgctgacctctctg R: tgtctccggtactcagtggac
FIBRONECTIN 1*	NM_002026.2	F: gccttcaagttcccctgttac R: aaccagaggctgactctctcc
SNAI-1^	NM_005985.4	F: ccctcttcctctccatacctg R: gcagaggacacagaaccagaa
SNAI-2^	NM_003068.5	F: tccagaccctggttgcttca R: tgacctgtctgcaaatgctct
CD44^	NM_00610.4 NM_001001389.2 NM_001001390.2 NM_001001391.2 NM_001202555.2 NM_001202556.2 NM_001202557.2	F: gatggagaaagctctgagcatc R: ttgctgcacagatggagttg
TGF-β*	NM_000660.7	F: cactctgagatgcagggactc R: tatcccccactaaagcaggtt

**Table 3:** Fibronectin expression in fibroblasts.

Culture Conditions	mRNA Fold Increase (2^−ΔΔCt^) 6 Days	mRNA Fold Increase (2^−ΔΔCt^) 12 Days
2D	1.23 ± 0.2	9.99 ± 1.6
3D	2.52 ± 0.4	19.2 ± 2.1

**Table 4:** TGF-β mRNA expression in spheroids after 21 days in culture.

Spheroids	mRNA Fold increase (2^−ΔΔCt^)
Breast	9.18 ± 0.87
Lung	14.02 ± 1.1

## Supplementary Materials

The following are available online at https://www.mdpi.com/2073-4409/8/11/1434/s1, Figure S1: FACS analysis of integrin receptors surface expression in lung and breast spheroids, Figure S2: Western blot and densitometric analysis analysis of EMT-related markers in Breast and in lung spheroids compared to adherent cells grown under differentiating conditions, Figure S3: FACS analysis of CD133 and CD44 surface expression in lung and breast spheroids.
