# Supplementary material for: Stem-Like Cancer Cells in a Dynamic 3D Culture System: A Model to Study Metastatic Cell Adhesion and Anti-Cancer Drugs
Source: Cells. 2019 Nov 13;8(11):1434. doi: 10.3390/cells8111434 (PMC6912649; doi:10.3390/cells8111434)
Supplement: Supplementary file 1 [file cells-08-01434-s001.pdf]

A

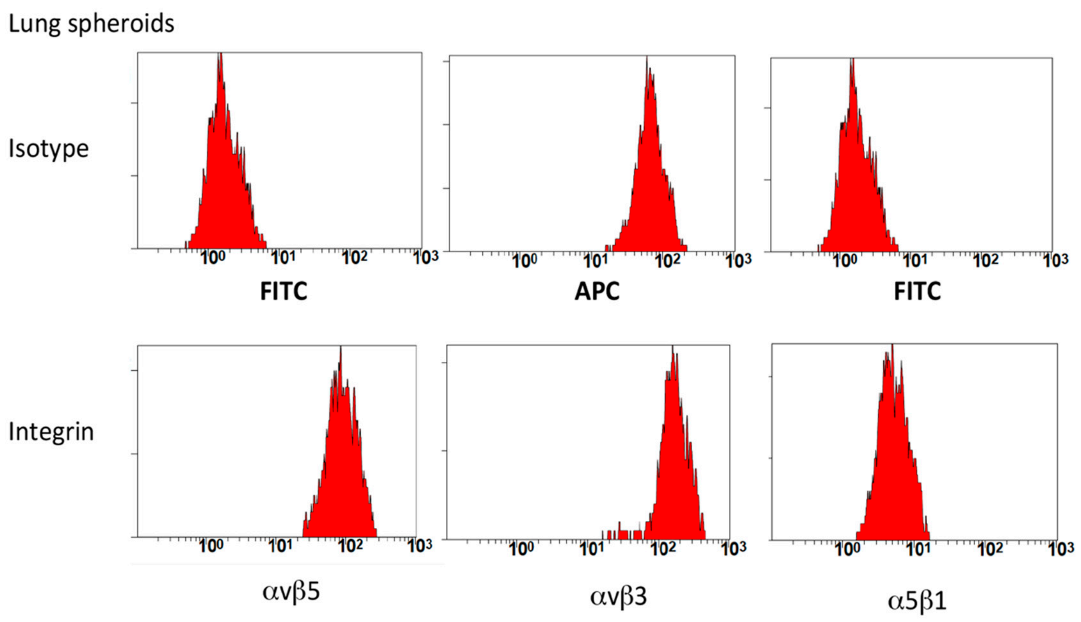

B

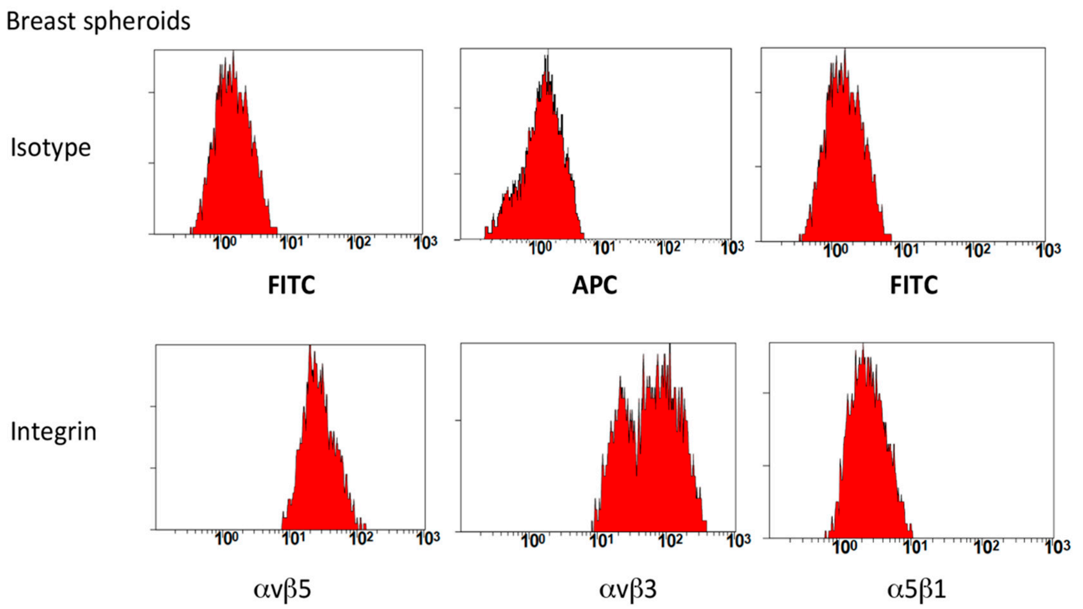

Figure S1. FACS analysis of integrin receptors surface expression in lung **(A)** and breast **(B)** spheroids. At least 10000 cells per each analysis were counted.

A

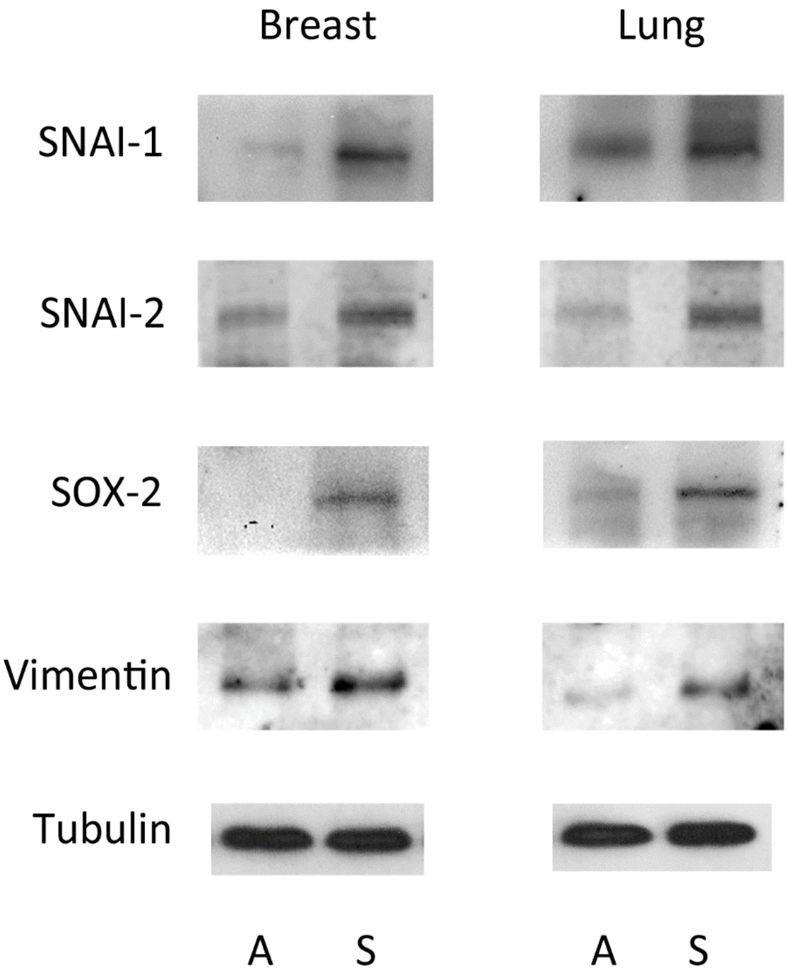

B

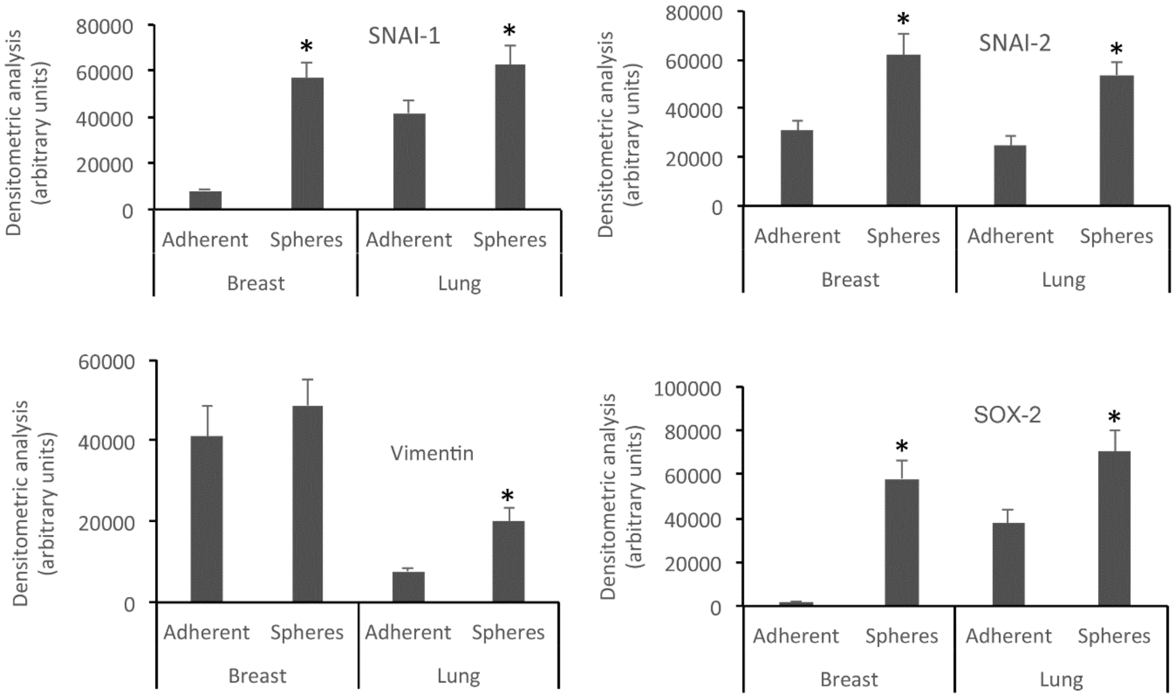

Figure S2. Western blot (**A**) and densitometric analysis analysis (**B**) of EMT-related markers in Breast and in lung spheroids compared to adherent cells grown under differentiating conditions. 35 g of cell extract were loaded in each lane. A: adherent cells; S: spheres. *p < 0,05 compared to adherent cells.

A

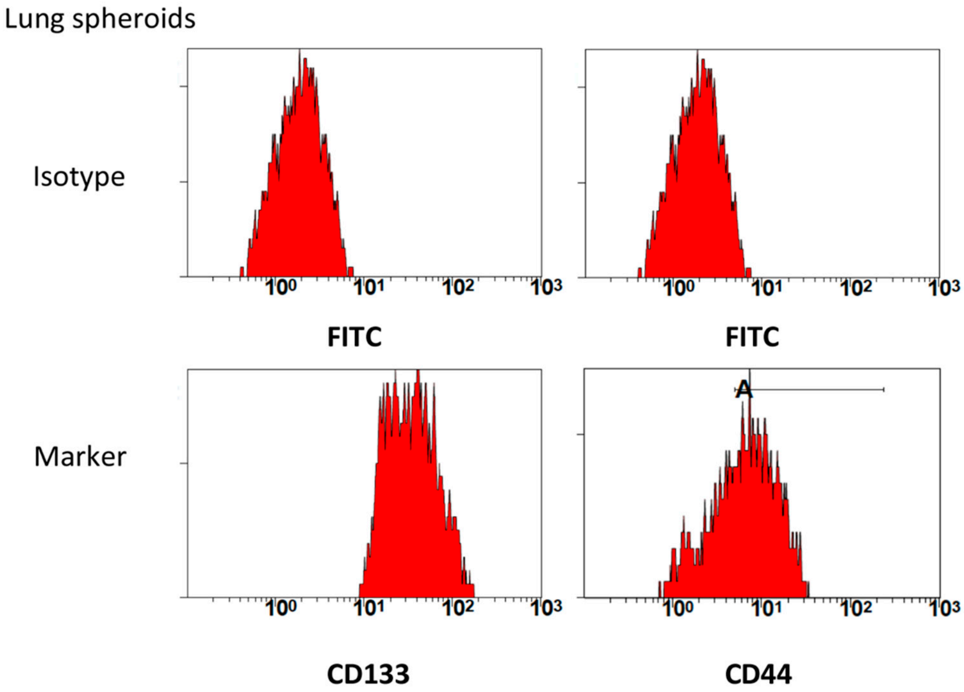

Lung spheroids
Isotype
Marker
FITC
FITC
CD133
CD44
A

B

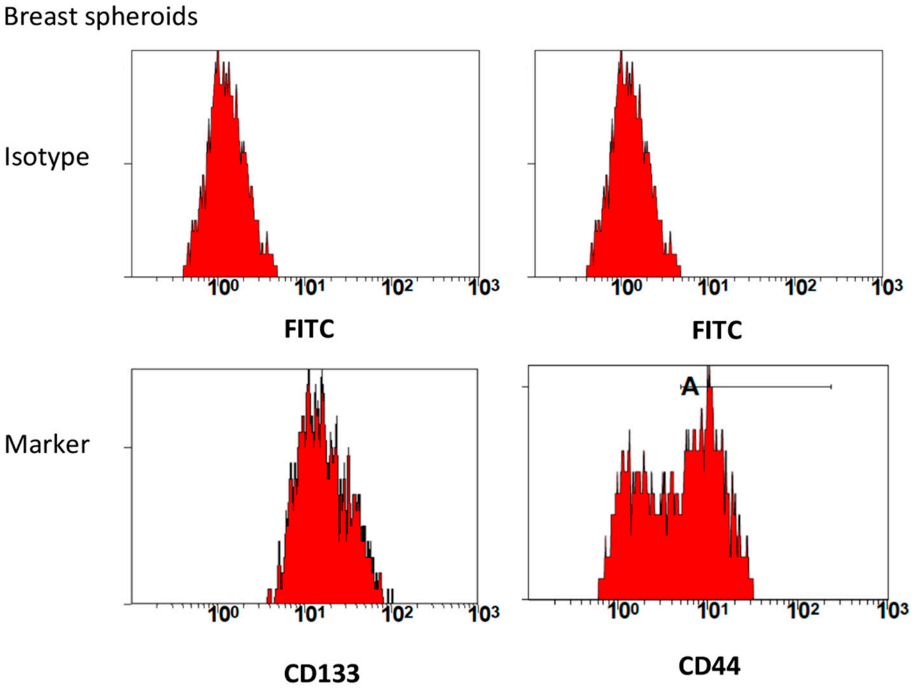

Breast spheroids
Isotype
Marker
FITC
FITC
CD133
CD44
A

C

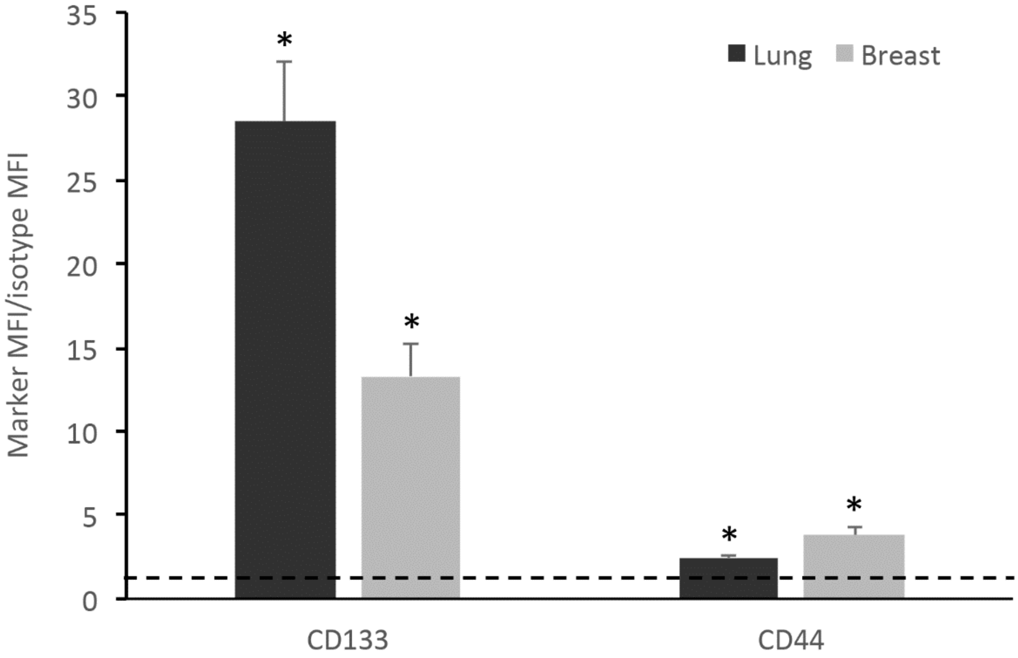

Figure S3. FACS analysis of CD133 and CD44 surface expression in lung **(A)** and breast **(B)** spheroids with graphic representation **(C).** At least 10000 cells per each analysis were counted. Dotted line: expression threshold = 1. *p < 0,05 compared to isotypic controls.
